# The Effect of Prostration (Sajdah) on the Prefrontal Brain Activity: A Pilot Study

**DOI:** 10.32598/bcn.9.10.195

**Published:** 2019-05-01

**Authors:** Fateme Yousefzadeh, Gila Pirzad Jahromi, Ehsan Mokari Manshadi, Boshra Hatef

**Affiliations:** 1. Medical Engineering, Alberta, Canada.; 2. Neuroscience Research Center, Baqiyatallah University of Medical Sciences, Tehran, Iran.

**Keywords:** Sajdah, EEG, Non-linear analysis, Brain activity, Gender

## Abstract

**Introduction:**

“Sajdah”, a prostration position, is part of Muslim daily prayers. It seems to have several effects on the brain and heart function. This study aimed to investigate the prefrontal brain activity after 10 seconds of Sajdah in the direction of Qibla (the direction that a Muslim prays) while putting the forehead on the ground.

**Methods:**

Three women and two men participated in this pilot study. Linear (absolute and relative power of θ (4–8Hz), α 1 (8–10 Hz), α 2 (10–12 Hz), β 1 (12–16 Hz), β 2 (16–20 Hz), β 3 (20–30 Hz), γ 1 (30–40 Hz), γ 2 (40–50 Hz) and non-linear features (approximate entropy, Katz fractal dimension, Petrosian fractal dimension, spectral entropy, and sample entropy) from Fps channel were calculated.

**Results:**

The relative β to γ band, approximate and sample entropy, Petrosian fractal dimension and mean of amplitude decreased in open eye state in women. While θ to γ bands in the closed eye state decreased after Sajdah in women. The absolute γ bands in closed eye state and relative β band in open eye state increased after Sajdah in men.

**Conclusion:**

The pilot study showed that 10 seconds of Sajdah has effects on brain activity and sometimes showed the opposite effect on genders.

## Highlights

- This pilot study assessed the EEG linear and non-linear features of the prefrontal region in the five healthy persons before and 20 seconds after the prostration (Sajdah) position.
- The results showed a significant change in the most relative power band of EEG frequency.
- Non-linear features such as fractal dimension and entropy in time decreased after Sajdah.
- The changes were more significant in women than men.

## Plain Language Summary

A 20-second Sajdeh showed a significant change in brain activity of the prefrontal region. It suggests that the complete Muslim praying (Namaz) has several effects that should be studied.

## 1. Introduction

Prayer is the most important daily duty of Muslims and comprises several acts and positions. During Sajdah, the subject is in prostrating position in the direction of Qibla (the direction that a Muslim prays) and some groups of Muslim community put their forehead on “Mohr”, which is made of dried clay. Rare studies showed the effect of Prayer on bio-signals like Electroencephalography (EEG) and Electrocardiography (ECG).

Doufesh et al. showed a significant decrease in heart rate and sympathetic activity during prayer and especially during Sajdah before and after prayer, especially in actual form (performing Muslim praying with all of nessasary conditions such as Wudu, Gibleh direction, positionings and especial Dhikrs (Doufesh, Ibrahim, Ismail, & Ahmad, 2013; Doufesh, Ibrahim, Ismail, & Wan Ahmad, 2014). They also reported the increase of relative α activity regardless of recitation in comparison with the resting position (Doufesh, Faisal, Lim, & Ibrahim, 2012; Doufesh et al., 2014). It is possible that the increase of α activity and absence of α blocking in the open eye are due to the higher state of calmness and focus as the head touches the ground. The amplitude of the γ band increased after prayer and this effect was significantly higher after listening to music (Ridzwan, Mahmood, Zakaria, & Ali, 2011).

The γ power during actual prayer was statistically higher than just staging prayer positions in the frontal and parietal regions in all stages, especially in the left hemisphere. Increased γ power during prayer, is possibly related to an increase in cognitive and attention processing (Doufesh, Ibrahim, & Safari, 2016).

Usually, prayer is compared with meditation. Many studies showed that some meditations had beneficial effects on the brain (Cahn & Polich, 2006; Ngo, 2013). During meditation, the increase of α band frequency primarily in the frontal region has been demonstrated (Cahn & Polich, 2006). On the other hand, some studies reported adverse effects during and after meditation (Shapiro, 1992). It must be noted that meditation techniques are often done in static posture such as sitting or lying supine, while prayer involves active physical movements.

EEG frequencies are associated with specific functions of the brain as an “electrophysiological signature.” For example, γ oscillations have been related to sensory processing, attention, action selection, conscious awareness and memory, and integrative function (van Wingerden, Vinck, Lankelma, & Pennartz, 2010). Also, δ oscillations have been correlated to motivation, reward processing, memory encoding, retrieval, and learning. The activity of θ band is associated with emotional arousal, fear conditioning and recognition memory (Knyazev, 2007). The α band has been associated with working memory functions and short-term memory. And β oscillations might be associated with the control of cognition (Engel & Fries, 2010; Neuner et al., 2014).

Studies using local field potentials, scalp, or cortical EEG recordings systematically revealed positive correlations between the power of the γ band (>30 Hz) and the BOLD (blood oxygenation level dependent) fluctuations at the same location. They reported the same correlation during cognitive, sensory and motor function at brain regions expected to be activated. The negative correlations exist between the power of the low-frequency ranges (α, β, and θ) and BOLD signals of those regions (Murta, Leite, Carmichael, Figueiredo, & Lemieux, 2015).

The studies about prayer show that the brain activity changes during the Sajdah more than other positions. Then the aim of this pilot study is to determine whether 10 seconds Sajdah as a part of prayer in the Qibla direction could induce any remained changes in the prefrontal area. On the other hand, the biological signals also had complex and chaotic pattern (Kaniusas, 2014) and the non-linear analysis of biological signals was more reliable than linear ones (Melillo, Bracale, & Pecchia, 2011). Then the brain activity was evaluated by non-linear analysis in addition to frequency and amplitude analysis. We hypothesize that Sajdah could change brain activity.

## 2. Methods

### 2.1. Study subjects

Two men (aged between 40 and 55 y) and 3 women (aged between 25 and 50 y) participated in the pilot study. We informed them about the study procedure and they signed the approval forms given by the Baqiyatallah University of Medical Sciences. All of the participants had the following characteristics: 1. No history of the psychological disorder based on DSM-5 guideline; 2. No report of surgery or trauma in the cranium and spine regions; 3. No history of taking regular neuropsychological medication; and 4. Always say their prayers regularly on time. They are right handed expect one of the male subjects.

### 2.2. Data acquisition

EEG signals were obtained from BioMed EEG system 32 channels (Made in Iran). Fp1 and Fp2 electrodes are based on the international 10.20 system attached to the scalp. The reference electrode was put in the Cz position and the ground electrode was attached to the right hand. The skin was cleaned with alcohol before electrode placing to reduce the skin impedance to 20 kΩ or less. Forty seconds of EEG was recorded with eyes open and eyes closed in the resting sitting position before Sajdah and then after 10 seconds of Sajdah in the Qibla direction. The test was done between 6 AM and 8 AM.

### 2.3. Data processing

EEG data were analyzed offline using MATLAB version R2014b. The obtained data were filtered through 0.2–48 Hz to remove any unwanted artifacts, including EOG (electrooculogram) and EMG (electromyogram). After artifact removal, the data were transformed to the average reference. Twenty-two linear features were extracted from signals in both states (eyes open and closed) before and after the Sajdah. The linear features were the absolute and relative power of frequency bands consisting of θ (4–8Hz), α 1 (8–10 Hz), α 2 (10–12 Hz), β 1 (12–16 Hz), β 2 (16–20 Hz), β 3 (20–30 Hz), γ 1 (30–40 Hz), γ 2 (40–50 Hz) and mean and variance of signal amplitude.

The relative features were calculated using the various band powers divided by the total power of the signal. The non-linear features were approximate entropy, Katz fractal dimension, Petrosian fractal dimension, sample entropy and spectral entropy. The Fp channels were preferred to study because the frontal region is contacted with the ground during the Sajdah.

### 2.4. Statistical analysis

The non-parametric Mann-Whitney U test was used to compare the EEG features before and after the Sajdah in separate groups in two different states. As a result of the small sample size and similar behavior in Fp1 and Fp2, they were considered together and mean of them was reported.

## 3. Results

### 3.1. The linear features of EEG

**Figure 1.**
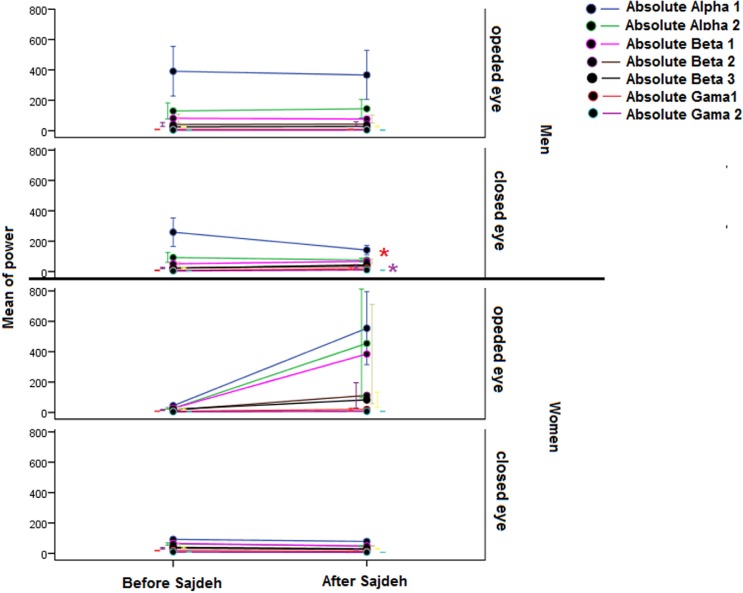
The plot showing the mean absolute power of EEG The γ 1 and γ 2 band significantly increased in men in the closed eye state. Significant change (P<0.05) was detected as a star sign with the same color of that in power band. θ band was deleted because of the high power and standard error.

**Figure 2.**
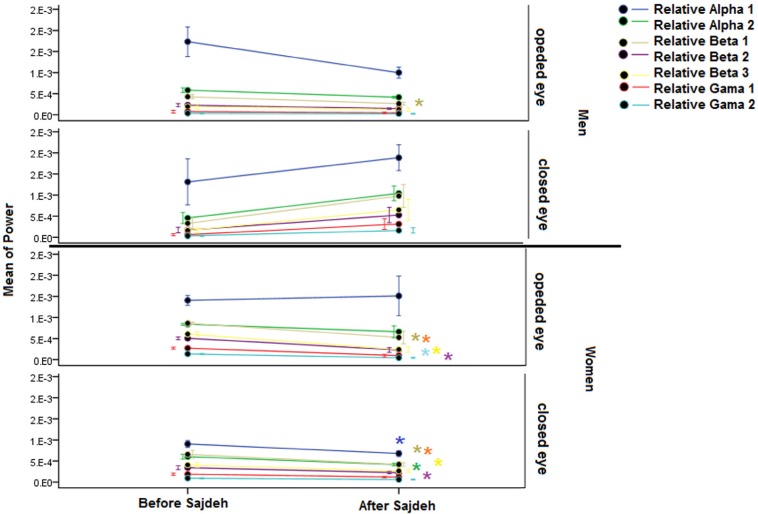
The plot showing the mean relative power of EEG with one standard error of α 1 to γ 2 A significant decrease was seen in the almost band in the β 2 to γ 2 band frequency in the opened eye recording and in the θ to γ 1 band frequency in the closed eye recording in the women. The θ band was deleted because of the high standard error. The significant change detected as star sign with the same color of that in power band.

The linear EEG features based on Mann-Whitney U test before and after Sajdah in open and closed eye states are presented in Table 1. The absolute power of band frequency did not show significant difference except an increase of the γ 1 and γ 2 after Sajdah in the state of closed eyes in men (Figure 1). Relative power showed a significant decrease in both states in women. The relative power from β 2 to γ 2 band frequency in the opened eye and from θ to γ 1 band frequency in the closed eye state decreased after Sajdah in women. Unlike women, the relative power band frequency tended to increase after the Sajdah in men in the closed eye state that was significant only in the β 1 band (Figure 2).

**Table 1.** P values of Mann-Whitney U test of EEG features before and after Sajdah in men and women, in the open and closed eye state

**Features**	Men (n=2)^*^		Women (n=3)^*^	

	**Opened Eyes**	**Closed Eyes**	**Opened Eyes**	**Closed Eyes**
Absolute θ	0.68	0.2	0.093	0.39
Absolute α 1	0.68	0.68	0.065	0.24
Absolute α 2	0.68	0.88	0.065	0.24
Absolute β 1	0.68	0.68	0.065	0.24
Absolute β 2	0.68	0.68	0.13	0.18
Absolute β 3	0.88	0.34	0.13	0.24
Absolute β1	0.34	0.029^*^	0.13	0.13
Absolute β 2	0.14	0.029^*^	0.13	0.31
Relative θ	0.68	0.68	0.58	0.004^*^
Relative α 1	0.2	0.34	0.58	0.041^*^
Relative α 2	0.11	0.11	0.065	0.041^*^
Relative β 1	0.02^*^	0.057	0.093	0.041^*^
Relative β 2	0.11	0.057	0.004^*^	0.041^*^
Relative β 3	0.34	0.057	0.004^*^	0.041^*^
Relative γ1	0.34	0.057	0.004^*^	0.041^*^
Relative γ 2	0.48	0.057	0.004^*^	0.13
Mean of amplitude	0.057	0.2	0.026^*^	0.81
Variation of amplitude	0.34	0.34	0.065	0.58
Approximate entropy	0.68	0.48	0.002^*^	0.041^*^
Katz fractal dimension	0.2	0.68	0.093	0.48
Petrosian fractal dimension	0.48	0.48	0.041^*^	0.065
Sample entropy	0.88	0.48	0.002^*^	0.093

* The significant data

**Figure 3.**
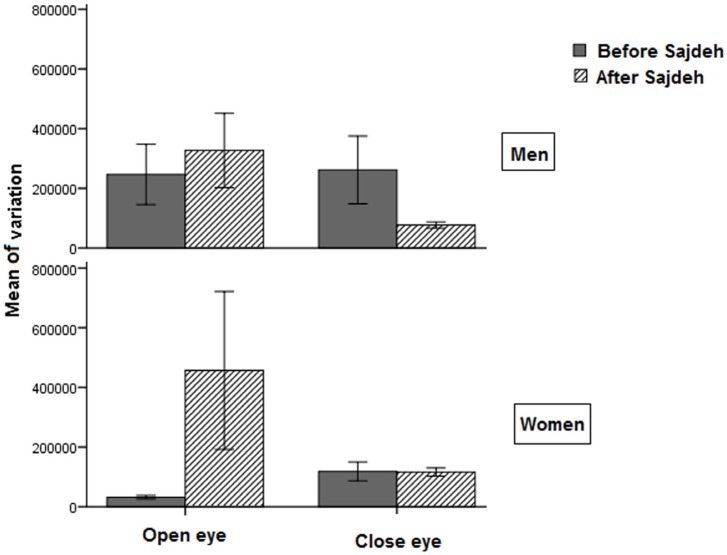
The plot showing the mean (standard error) variation of EEG amplitude in the open and closed eye recording No significant difference was seen.

The mean value of EEG amplitude changed after the Sajdah in women in the open eye recordings. But the variation of EEG amplitude did not show significant difference although there was an increasing trend in the women, especially in the open eye recording (Table 2, Figure 3).

**Table 2.** The Mean**±**SD of the EEG amplitude in the open and closed eye recording in men and women, before and after the Sajdah

**Variables**	**Opened Eye**				**Closed Eye**			

	**Men (n=2)**		**Women (n=3)**		**Men (n=2)**		**Women (n=3)**	

	**Before**	**After**	**Before**	**After**	**Before**	**After**	**Before**	**After**
Mean of amplitude	−4.2**±**0.3	−0.45±1.4	0.73±0.2	−0.84±0.4^*^	1.7±1	−1.5±2	−1.7±2.4	−0.22±0.9

* Shows significant difference in the effect of a Sajdah in women in the open eye recording (P<0.003).

### 3.2. The non-linear features of EEG

**Figure 4.**
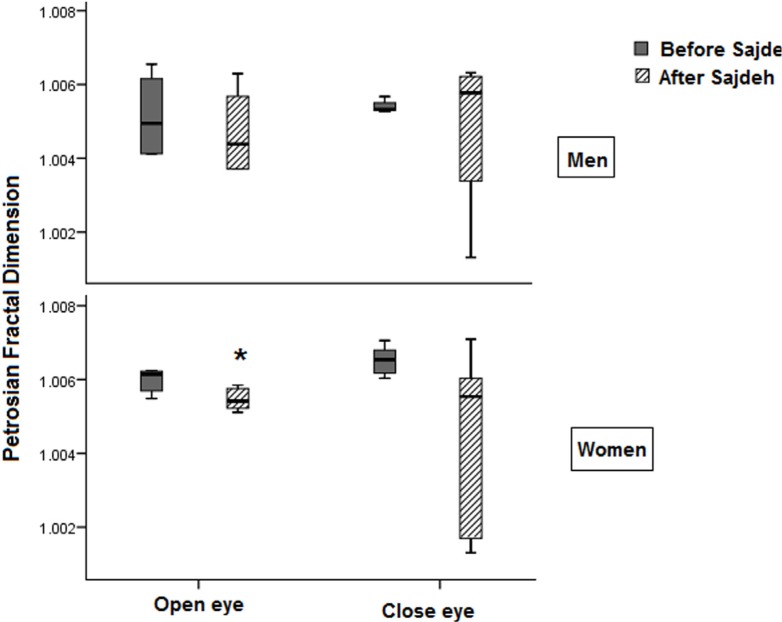
The plot showing the Petrosian fractal dimension affected by Sajdah, especially in women in the eye open recording ^*^ P<0.05.

**Figure 5.**
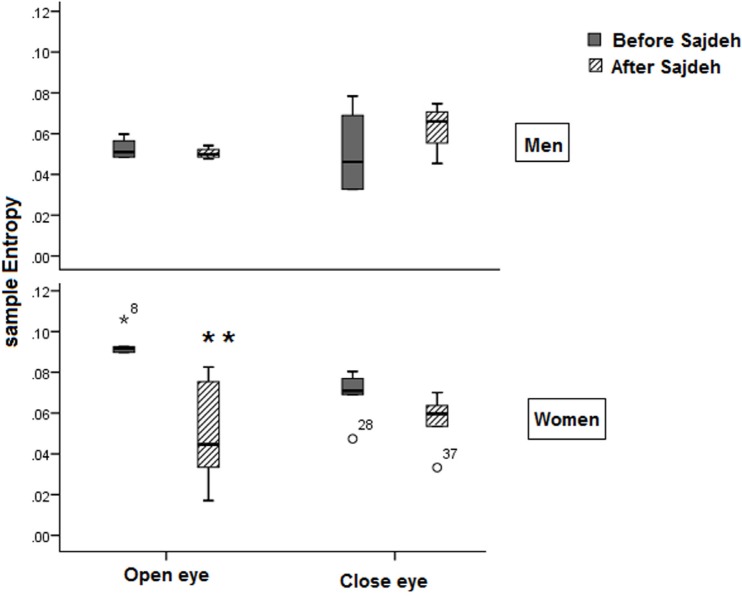
The plot showing approximate entropy decrease after Sajdah in the women (n=3) ^*^ P<0.05; ^**^ P<0.003

**Figure 6.**
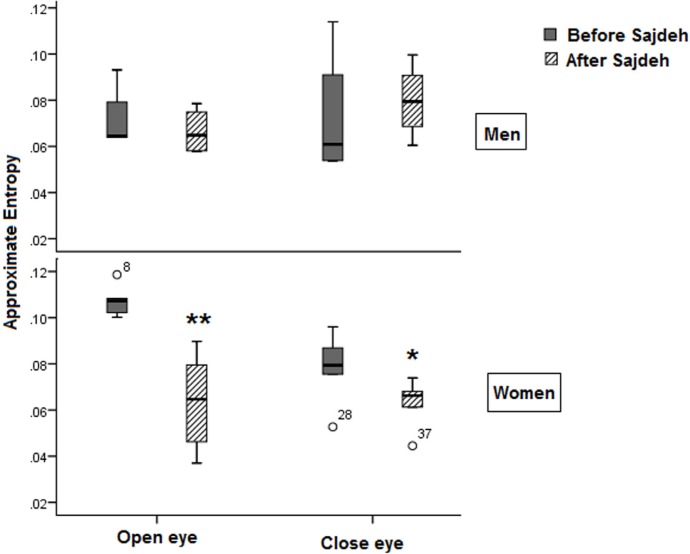
The plot showing sample entropy decrease after Sajdah in women significantly in the open eye recording ^*^ P<0.05; ^**^ P<0.003

The fractal non-linear features of EEG such as Katz fractal dimension and Petrosian fractal dimension showed that the Sajdah decreased significantly the fractal dimension of EEG signals in the prefrontal region in women in open eye state (Figure 4). Approximate entropy and sample entropy decreased significantly in the open eye state in women (Figures 5 and 6).

The Mann-Whitney U test was also used to compare the EEG features between men and women before and after Sajdah. The results showed that gender differences in the EEG features diminished after Sajdah in the open eye state and in the non-linear features in both states of eyes. It means that the trend of changes in the two genders was different after the Sajdah that was significantly evident in the open eye recording (Table 3).

**Table 3.** The P values of Mann-Whitney U test in men and women before and after the Sajdah in the open and closed eye

**Features**	**Opened Eyes**		**Closed Eyes**	

	**Before Sajdah**	**After Sajdah**	**Before Sajdah**	**After Sajdah**
Absolute θ	0.11	0.76	0.14	0.038
Absolute α 1	0.067	0.91	0.25	0.17
Absolute α 2	0.067	1	0.76	0.17
Absolute β 1	0.11	1	0.35	0.17
Absolute β 2	0.11	1	0.11	0.25
Absolute β 3	0.47	0.27	0.11	0.47
Absolute γ 1	0.47	0.27	0.01	0.61
Absolute γ 2	0.47	0.35	0.01	1
Relative θ	0.76	0.91	0.01	0.01
Relative α 1	0.47	0.76	0.91	0.01
Relative α 2	0.01	0.11	0.25	0.01
Relative β 1	0.01	0.61	0.11	0.01
Relative β 2	0.01	0.61	0.11	0.038
Relative β 3	0.01	0.25	0.03	0.038
Relative γ1	0.01	0.35	0.03	0.17
Relative γ 2	0.01	0.61	0.03	0.25
Mean of amplitude	0.01	0.91	0.35	0.61
Variation of amplitude	0.01	0.91	.76	0.067
Approximate entropy	0.01	1	0.61	0.17
Katz fractal dimension,	0.01	0.47	0.11	0.11
Petrosian fractal dimension	0.47	0.25	0.01	0.76
Sample entropy	0.01	0.76	0.25	0.35

## 4. Discussion

The aim of this pilot study was to measure the linear and non-linear features of brain activity in the prefrontal region before and after one Sajdah (for 10 seconds), as a part of Muslim daily prayer. In spite of the small sample size, some clearly significant effects of the Sajdah were seen especially in women at the open eye recording. The results showed the decrease of the relative power of bands, especially β and γ oscillations in both open and closed eye recording and decrease of entropy and Petrosian fractal dimension as a non-linear feature of EEG in the open eye state after the Sajdah in women. Increase of the γ power during and after prayer had been demonstrated before. But the participants were men (Doufesh et al., 2016; Ridzwan et al., 2011).

Our study evaluated the women too and showed the opposite effect of one Sajdah to relative γ band in women but not in men. On the other hand, the brain activity after one Sajdah did not cover the effect of prayer and they could be different and not comparable. Therefore the current results confirmed the previous results in men with respect to the absolute power of the γ band.

The non-linear features showed that the entropy and fractal dimension of EEG signals as indices of signal complexity (Ahmadi, Ahmadlou, Rezazade, Azad-Marzabadi, & Sajedi, 2013; Micheloyannis, Vourkas, Bizas, Simos, & Stam, 2003) decreased in women. Ten seconds of Sajdah like any other cognitive tasks (Corsi-Cabrera, Ramos, Guevara, Arce, & Gutierrez, 1993; Kober, Reichert, Neuper, & Wood, 2016) or exposure to electromagnetic field (Papageorgiou, Nanou, Tsiafakis, Capsalis, & Rabavilas, 2004) has inverse effect in brain activity of men and women which could be the result of gender structural differences (Ahmadi et al., 2013; Corsi-Cabrera et al., 1993; Kober et al., 2016; McGivern et al., 1998).

The effects of sex steroids in the human brain may play some role in explaining these differences. The sex steroids interact with neurotransmitters and other hormones such as the oxytocin-vasopressin system in the brain that regulates the brain function (Nguyen, Ducharme, & Karama, 2016). Several studies show that some cognitive abilities are higher in women and some of them are advanced in men (Halpern & LaMay, 2000; Li, 2014; McGivern et al., 1998). The interesting finding shown in Table 3 indicates that the significant baseline linear and non-linear features of EEG between genders decreased or changed after the Sajdah.

The increase of γ power that was seen after the Sajdah has been related to increased activity of the frontal node of the Default Mode Network (DMN), the medial prefrontal cortex (Berkovich-Ohana, Glicksohn, & Goldstein, 2012), and the cognitive activity (van Wingerden et al., 2010). The increase of α oscillation was related to working memory (Neuner et al., 2014) and θ oscillation to emotional processing (Knyazev, 2007). The event-related potentials studies concluded that meditation can increase attention and enhance emotional control that matched with an increase of θ and α oscillations in meditation (Singh & Telles, 2015).

The results of studies in meditation or prayer have been reported in the men or mixed gender group (Cahn & Polich, 2006; Doufesh et al., 2012; Doufesh et al., 2014; Doufesh et al., 2016; Singh & Telles, 2015) and there was no study that measured the effect of meditation or prayer in the brain activity with interaction gender. Whenever the Sajdah as a part of Muslim praying showed some changes but it could not be referred to the effect of the completed prayer.

Some researchers believe that prayer is a type of meditation. But there are significant differences between them in terms of the action and effects. Prayer involves physical movements with specific and fixed saying and pattern but meditation often involves static position without saying in several patterns. The meditation research with expanding the methodological paradigm of cultural setting as the place of meditator, the particular practice, and the state of consciousness of mediators showed several states and trait effects on brain activity, especially increased power of low frequency such as θ and α bands and decrease of γ oscillations over the frontal and midline regions (Berkovich-Ohana et al., 2012; Cahn & Polich, 2006; Kaur & Singh, 2015; Thomas & Cohen, 2014). Whereas prayer increased both the power of α and γ band (Doufesh et al., 2012; Doufesh et al., 2014; Doufesh et al., 2016). Therefore the comparison of them is not always appropriate.

No study has measured non-linear features of EEG in the whole of prayer or meditation or a part of them. Whereas our results showed that Sajdah had a significant effect on the complexity of the EEG signal. The decrease of sample and approximate entropy and Petrosian fractal dimension were detected after the Sajdah in the women, especially in the open eyes state. The signal complexity of EEG is correlated to high-frequency oscillations, especially γ band. (Micheloyannis et al., 2003; Murta et al., 2015; Pravitha, Sreenivasan, & Nampoori, 2005).

On the other hand, the decrease in complexity was shown in inhibition control as an adaptive ability of brain (Huang, Tseng, & Liang, 2015). Lower neural complexity does not always indicate the declines in information processing and cognitive function (McDonough & Nashiro, 2014). The decrease of γ power and signal complexity in women might be the result of shifting of the cognitive processing from sensory and motor processing to inhibition control dominance and decreased activity of the frontal node of DMN. The limitations of the study were the small sample size, limited recorded electrodes, and not recording during Sajdah or in the real prayer and assessment of the whole prayer.

The pilot study showed that 10 seconds of Sajdah in the direction of Qibla while putting the forehead on Mohr significantly influenced the brain activity on the prefrontal region. Sajdah had opposite effects on different genders. In women, the power bands, especially γ and β oscillation and the complexity of signals decreased, especially in open eye recording. Whereas the effect of Sajdah showed an increase in the absolute power of β or γ frequency band of EEG in men.

The genders showed significant baseline differences in the linear and non-linear features of brain activity in the prefrontal region after Sajdah. The findings of the pilot study need to be further evaluated by other studies with enough sample size and in real prayer. The Sajdah is only one part of the prayer which showed significant improvement in brain activity. The Muslims are supposed to do it five times a day. Thus the effect of prayer to maintain and improve brain activity and mental health is critical and needs to be studied more in the future.
